# On realized serial and generation intervals given control measures: The COVID-19 pandemic case

**DOI:** 10.1371/journal.pcbi.1008892

**Published:** 2021-03-29

**Authors:** Andrea Torneri, Pieter Libin, Gianpaolo Scalia Tomba, Christel Faes, James G. Wood, Niel Hens

**Affiliations:** 1 Centre for Health Economic Research and Modelling Infectious Diseases, University of Antwerp, Antwerp, Belgium; 2 Interuniversity Institute of Biostatistics and statistical Bioinformatics, Data Science Institute, Hasselt University, Hasselt, Belgium; 3 Artificial Intelligence Lab, Department of Computer Science, Vrije Universiteit Brussel, Brussels, Belgium; 4 KU Leuven, Department of Microbiology and Immunology, Rega Institute for Medical Research, University of Leuven, Leuven, Belgium; 5 Department of Mathematics, University of Rome Tor Vergata, Rome, Italy; 6 School of Public Health and Community Medicine, UNSW Sydney, Sydney, Australia; University of Notre Dame, UNITED STATES

## Abstract

The SARS-CoV-2 pathogen is currently spreading worldwide and its propensity for presymptomatic and asymptomatic transmission makes it difficult to control. The control measures adopted in several countries aim at isolating individuals once diagnosed, limiting their social interactions and consequently their transmission probability. These interventions, which have a strong impact on the disease dynamics, can affect the inference of the epidemiological quantities. We first present a theoretical explanation of the effect caused by non-pharmaceutical intervention measures on the mean serial and generation intervals. Then, in a simulation study, we vary the assumed efficacy of control measures and quantify the effect on the mean and variance of realized generation and serial intervals. The simulation results show that the realized serial and generation intervals both depend on control measures and their values contract according to the efficacy of the intervention strategies. Interestingly, the mean serial interval differs from the mean generation interval. The deviation between these two values depends on two factors. First, the number of undiagnosed infectious individuals. Second, the relationship between infectiousness, symptom onset and timing of isolation. Similarly, the standard deviations of realized serial and generation intervals do not coincide, with the former shorter than the latter on average. The findings of this study are directly relevant to estimates performed for the current COVID-19 pandemic. In particular, the effective reproduction number is often inferred using both daily incidence data and the generation interval. Failing to account for either contraction or mis-specification by using the serial interval could lead to biased estimates of the effective reproduction number. Consequently, this might affect the choices made by decision makers when deciding which control measures to apply based on the value of the quantity thereof.

## Author summary

The generation and serial intervals are epidemiological quantities used to describe and predict an ongoing epidemic outbreak. These quantities are related to the contact pattern of individuals, since infection events can take place if infectious and susceptible individuals have a contact. Therefore, intervention measures that reduce the interactions between members of the population are expected to affect both the realized generation and serial intervals. For the current COVID-19 pandemic unprecedented interventions have been adopted worldwide, e.g. strict lockdown, isolation and quarantine, which influence the realized value of generation and serial intervals. The extent of the effect thereof depends on the efficacy of the control measure in place, on the relationship between symptom onset and infectiousness and on the proportion of infectious individuals that can be detected. To get more insight on this, we present an investigation that highlights the effect of quarantine and isolation on realized generation and serial intervals. In particular, we show that not only their variances but also their mean values can differ, suggesting that the use of the mean serial interval as a proxy for the mean generation time can lead to biased estimates of epidemiological quantities.

## Introduction

On December 31 2019, the city of Wuhan (Hubei province, China) reported an outbreak of atypical pneumonia caused by a novel coronavirus, later on named SARS-CoV-2 [1]. Viral and epidemiological characteristics of COVID-19 outbreaks such as asymptomatic transmissions [2, 3] and undiagnosed individuals [4] hastened the spread of the disease, resulting in a global pandemic. In response to this threat, several countries adopted drastic and unprecedented control measures such as national and regional lockdown in which diagnosed or traced individuals were confined in isolation or quarantine [5]. Concurrently, researchers started to estimate key epidemiological quantities such as the incubation period, the serial interval, the generation time and the reproduction number (see e.g. [3, 6–11]). It is important to note that most of these epidemiological determinants were inferred in situations for which different intervention strategies were in place, possibly affecting the resulting estimates. More precisely, control measures such as quarantine or isolation of infectives intervene in reducing the social contacts of such individuals, and consequently in decreasing their probability of transmission after diagnosis. These interventions lead to a delay of the epidemic peak [12] and to a decrease of the effective reproduction number [13]. Therefore, it is reasonable to think that other epidemic characteristics are also affected by public health interventions.

A key quantity in epidemic modelling is the generation time or generation interval, i.e. the time difference between the infection times of an infector-infectee pair [14]. In particular, the generation time can be used to compute the basic and effective reproduction numbers by means of the Lotka-Euler equation [15]. Often, the generation time is approximated by the serial interval [16], i.e. the time difference between the onset of symptoms of an infector-infectee pair, and, by doing so, estimates of the basic reproduction number for the COVID-19 epidemic have been computed [10, 11]. Britton and Scalia Tomba [17] showed that replacing the generation time with the serial interval could lead to an understimation of the (basic) reproduction number. In fact, while they have the same mean, the variance of the former is larger than that of the latter quantity. In addition, Park et al. discuss that if asymptomatic and symptomatic individuals have different generation time distributions, estimation of the reproduction number is biased if performed using only data for the symptomatic population [18]. Non-pharmaceutical interventions such as isolation, which decrease the social interactions of diagnosed individuals, affect the realized generation time distribution of only diagnosed individuals, likely causing the aforementioned bias. Moreover, Park et al. showed how the serial interval can be used to estimate the basic reproduction number [19]. However, in their analysis, they did not consider the effect of control measures on the realized serial and generation intervals. Recently, Ali et al. investigated the relationship between serial interval and non-pharmaceutical interventions, reporting a modification of serial interval distributions as a function of control measures in Chinese data [20]. In particular, they showed that the mean serial interval decreases when control measures are more stringent. Similarly, Sun et al. reported a contraction of the serial and generation intervals when different intervention measures were in place [21]. However, the quantities thereof could contract differently, resulting in a difference between their mean values. In fact, the relationship between serial and generation interval has not yet been explored in the setting of control measures.

In this work we highlight three characteristics of the COVID-19 pandemic dynamic that affect the mean and the variance of the serial and generation intervals. First, the efficacy of control measures, determined by the starting timing of quarantine and isolation relative to exposure and by the magnitude of the contact rate reduction. Second, the presence of an undiagnosed population, of which the individuals escape diagnosis, such that it is not affected by the considered interventions. Third, the relationship between infectiousness, incubation period and time at which interventions take place during the infectious period. After presenting a theoretical description of the effect of control measures on the expected serial and generation intervals, we set up a simulation study to gain insights into the determinants that affect the realized serial and generation intervals in the presence of control measures.

## Methods

Throughout the manuscript, when referring to a control measure we allude to a non-pharmaceutical intervention occurring during the infectious period of an infective individual that reduces the contact rate of the diagnosed infected individual. We assume that control measures are applied only to diagnosed individuals and that these take place after symptom onset. We define the time to diagnosis, to be the time from the onset of symptoms to the start of the intervention measure. Furthermore, we define a generation to be an infectious event caused by a specific infector. As described in Champredon and Dushoff [22], we define a realized generation to be an infection event that takes place during the epidemic. While considering a realized generation, we account for the realized generation interval as the time difference between the infection times of such an infection pair. The realized serial intervals refers instead to the time difference between their times of symptom onset. We consider serial intervals only for the infector-infectee pairs in which both individuals are diagnosed, to represent the currently applied inference methodologies [8, 23, 24]. Instead, the generation time is computed for all the transmission events that take place during the simulated outbreak. In the following, we first present a heuristic derivation of the relationship between control measures, serial interval, generation time and incubation period. Afterwards, we present a simulation study that highlights the effect of control measures on the mean and variance of realized serial and generation intervals.

### Expected serial intervals conditioned on control measures

We consider a homogeneous population and let *i* be an infectious individual that makes at least one generation. Moreover, we assume *i* to show symptoms sooner or later in his or her infectious period. To this individual, we can associate a pair (*G*^*i*,*j*^, Σ^*i*^) where the first term is one of his or her realized generation times and the second one describes the incubation period length. Let *j* be the individual infected by *i* at the generation time *G*^*i*,*j*^. Following Britton and Scalia Tomba [17] we can write the serial interval, *S*^*i*,*j*^, relative to the generation *G*^*i*,*j*^ as:

$$\begin{array}{c} S^{i,j}=G^{i,j}+\Sigma^{j}-\Sigma^{i}, \end{array}$$ (1)

and $E\left[S^{i,j}\right]=E\left[G^{i,j}\right]$ holds following the homogeneity assumption $E\left[\Sigma^{j}\right]=E\left[\Sigma^{i}\right]$.

We now consider a control measure that limits the infectiousness after symptom onset, allowing generations to be realized only in the presymptomatic phase. Conditioned on this, the couple (*G*^*i*,*j*^, Σ^*i*^) assigned to *i* satisfies *G*^*i*,*j*^ < Σ^*i*^ and the relative serial interval results in:

$$\begin{array}{c} E\left[S^{i,j}|G^{i,j}<\Sigma^{i}\right]=E\left[G^{i,j}|G^{i,j}<\Sigma^{i}\right]+E\left[\Sigma^{j}\right]-E\left[\Sigma^{i}|G^{i,j}<\Sigma^{i}\right], \end{array}$$ (2)

assuming that Σ^*j*^ is independent from the event *G*^*i*,*j*^ < Σ^*i*^. We here consider joint distributions of (*G*^*i*,*j*^, Σ^*i*^) for which:

$$\begin{array}{c} E\left[G^{i,j}|G^{i,j}<\Sigma^{i}\right]<E\left[G^{i,j}\right]. \end{array}$$ (3)

While theoretically this result does not hold in general, Eq 3 is satisfied by the simulation model and the control measures considered in this work. Precisely, this equation expresses that control measures reduce the observed generation times, since generations that normally would take place later on during the infectious period are less likely to occur. In addition, this inequality is expected to hold for most of the epidemic models and intervention strategies that reduce the infectiousness.

Let us assume that control meausures affect the incubation period of infectors such that have on average a larger incubation period compared to the unconditional case:

$$\begin{array}{c} E\left[\Sigma^{i}\right]-E\left[\Sigma^{i}|G^{i,j}<\Sigma^{i}\right]\leq 0. \end{array}$$ (4)

When this relation holds, from Eqs 3 and 4 it follows that the serial interval contracts in presence of interventions:

$$\begin{array}{c} E\left[S^{i,j}|G^{i,j}<\Sigma^{i}\right]<E\left[G^{i,j}\right]+E\left[\Sigma^{j}\right]-E\left[\Sigma^{i}\right]=E\left[S^{i,j}\right]. \end{array}$$ (5)

Furthermore, from Eq 4 and the homogeneity assumption $E\left[\Sigma^{i}\right]=E\left[\Sigma^{j}\right]$ the realized serial interval results to be shorter or equal, on average, to the realized generation time:

$$\begin{array}{c} E\left[S^{i,j}|G^{i,j}<\Sigma^{i}\right]\leq E\left[G^{i,j}|G^{i,j}<\Sigma^{i}\right]. \end{array}$$ (6)

We notice that the above inequality is strict when the inequality in Eq 4 is strict. Differently, when interventions facilitate individuals with shorter incubation period to spread the infectious disease, the same derivation can not be performed and the realized serial interval could potentially become larger when control measures are in place. In addition, in this case the realized serial interval is on average larger than the realized generation time, i.e.:

$$\begin{array}{c} E\left[S^{i,j}|G^{i,j}<\Sigma^{i}\right]>E\left[G^{i,j}|G^{i,j}<\Sigma^{i}\right]. \end{array}$$ (7)

Therefore, except when the equality holds in Eq 4, control measures cause a deviation from the mean serial to the mean generation intervals.

This derivation highlights the importance of considering the bivariate distribution of (*G*^*i*,*j*^, Σ^*i*^) to properly assess the difference between serial and generation intervals. Yet, it is not straightforward to characterize theoretically the properties that (*G*^*i*,*j*^, Σ^*i*^) need to satisfy to fulfill the Eq 4 or Eq 7. In a simple case, if *G*^*i*,*j*^ and Σ^*i*^ are independent and with overlapping support, both Eqs 3 and 4 hold. Though, this assumption does not make biological sense and more realistic relationships should be inferred from suitable data.

The above derivation accounts for control measures that take place at symptom onset, but the same description holds when interventions take place at a random time *Z* during the infectious period. In this case, the generation time, serial interval and incubation period will be conditioned on the event *G*^*i*,*j*^ < *Z*. To get more insight on this, we investigate the effect of control measures on the realized incubation period of infectors in a simulation study, consequently highlighting their impact on the difference between mean realized serial and generation intervals.

#### Undiagnosed population

We define undiagnosed individuals to be asymptomatic or mild cases who do not show symptoms severe enough to be detected. According to this definition, this class of individuals is not affected by the control measures, since these apply only to the diagnosed persons who can be detected only after symptom onset. Therefore, their generation time distribution is independent from the intervention strategy in place. When diagnosed and undiagnosed individuals are equally infectious, the expected generation time conditional on the control measure is larger for individuals who escape detection:

$$\begin{array}{c} E\left[G^{u}\right]=E\left[G^{d}\right]>E\left[G^{d}|G^{d}<\Sigma^{i}\right], \end{array}$$ (8)

where *G*^*u*^, *G*^*d*^ indicate, respectively, the generation time of undiagnosed and diagnosed individuals. In this framework, the serial interval is not computed for infections generated by undiagnosed individuals, since either they are not showing symptoms or symptoms were not related to the considered infectious disease. Therefore, undiagnosed persons contribute only in increasing the generation time. We note that in practice, the intrinsic generation time might differ by symptom status and this would alter the degree to which the observed generation interval is biased by non-detection of undiagnosed cases.

### Simulation study

The simulation framework extends on the stochastic epidemic model presented in Torneri et al. [25] used to represent the effect of control measures that limit infectiousness, such as quarantine, isolation and administration of antivirals. Briefly, contacts between different members of the population are generated and such contacts, when realized between an infectious and a susceptible individual, result in infection events according to a probability value that depends on the viral progression within the infectious individual. Such a probability is a combination of an infectivity measure, that describes the distribution of the viral progression over time, and of an infectivity term, that quantifies the number of possible generations infectives realize. In this construction, the reproduction number is approximated with the number of effective contacts that infective individuals make during their infectious period. The infectivity measure is set to represent the viral shedding evolution of SARS-CoV-2. This curve represents the viral progression from the time of infection until the time infectives stop shedding the virus. Furthermore, this curve is scaled among different infectious individuals to acknowledge individual heterogeneity in the viral progression over time. More details on this can be found in S1 Appendix.

Infectives are classified as either undiagnosed or diagnosed. In the former class we include asymptomatic and symptomatic cases with symptoms that are not severe enough to be detected. Individuals of the latter class are assumed to experience symptoms that can be detected and linked to the ongoing infection. Diagnosis is assume to take place at the time of symptom onset, representing the current high awareness of the COVID-19 pandemic. When diagnosis take place, infectives are immediately quarantined or isolated, showing, respectively, mild or severe symptoms. Quarantine and isolation last until the infectious individual recovers completely from the infection and can not spread the disease anymore. If infectives are diagnosed after recovery, no interventions are applied to them. Isolation and quarantine are implemented via a reduction in the contact rate. In the former case, the contact rate is reduced to 5% of the regular contact rate. According to this, isolated individuals still have a small probability of spreading the infection. In the home quarantine setting, infectious individuals are assumed to make, on average, 3 contacts a day, instead of 12. This reduction in the contact rate is set to represent the mean number of contacts that infectious individuals can possibly make during quarantine with their household members. In the simulation, each contact is simulated with a random member of the population and therefore represent and approximation of home quarantine, where contacts are realized with the same household members. The daily contact rates discussed above are set according to values provided by the Socrates tool [26] for the Belgian population.

#### Baseline scenario and summary measures

**Fig 1:**
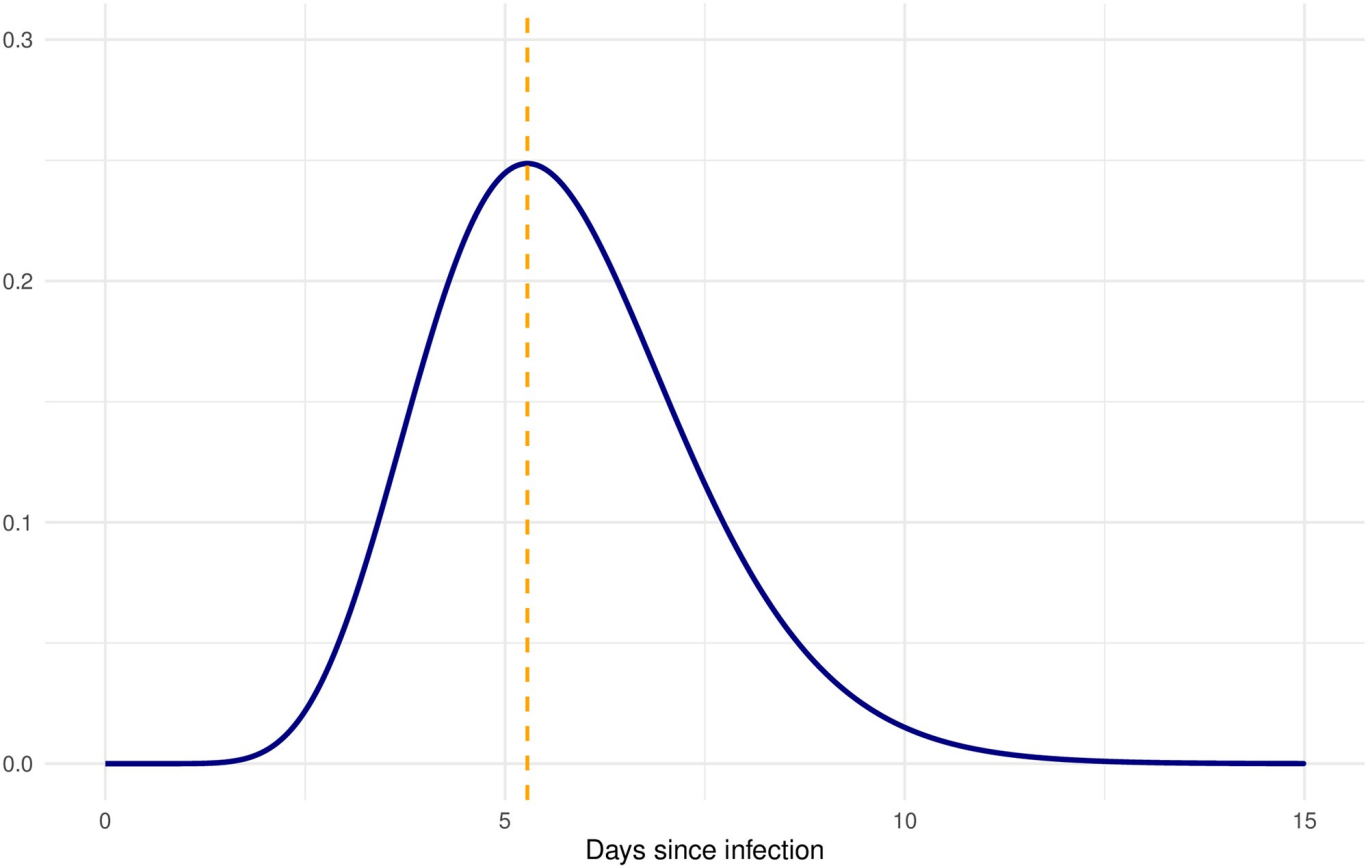
Infectivity measure. Infectivity Measure over the infectious period (blue line) and day of symptom onset (dashed orange line).

The baseline scenario is set to best represent the current COVID-19 pandemic (Table 1). Each symptomatic individual is assumed to show symptoms at the peak of the infectiousness, as indicated by recent literature findings [21, 27]. In our framework the infectiousness is expressed as the combination of a viral component, i.e. infectivity and infectivity measure (see section 2.2), and of the contact rate. Furthermore, with infectiousness profile we indicate the temporal evolution of the infectiousness over the infectious period. Since in our setting the contact rate only decreases when infectious individuals are diagnosed, because of the effect of quarantine and isolation, the peak of the infectivity measure corresponds to the peak of the infectiousness. The value of the reproduction number depend on whether infectives show symptoms severe enough to be diagnosed and if so on the severity of the experienced symptoms. Severe cases are assumed to have a larger reproduction number as compared to mild cases, since a larger mean viral load has been observed for these infectives [28]. Several different studies are reported in literature on viral load and viral shedding observation for the SARS-CoV-2 pathogen [27–32] indicating the uncertainty on this value. In particular, these investigations do not always look at the shedding of live virus, which to date was not detected yet after 9 days from infection [33] and it reasonably is the proper proxy to use in models for transmission. Therefore, based on these studies, we define an infectivity measure that peaks at symptom onset and lasts 10 days, on average. In addition, the infectivity measure has an initial plateau with value zero, accounting for the latent phase. We show this infectivity measure in Fig 1. Moreover, as result of the chosen setting, the incubation period is on average of 5.2 days, in line with other results from literature [7, 24]. However, its distribution is less skewed and more concentrated around the mean (Fig B in S1 Appendix). This is a result of the assumptions made on the functional form of the infectivity measure, how such a curve is scaled among infectious individuals together with the assumption that symptom onset at when the infectivity measure peaks.

**Table 1:** Model parameters—Baseline scenario.

Name	Mean Value (sd)	Distribution	Reference
Time to diagnosis	Upon Symptom	NA	Assumed
Reproduction number:	2.3		[7, 24]
- Undiagnosed ($R_{0}^{u}$)	1.375 (0.94)	NegBinom	[4]
- Mild ($R_{0}^{m}$)	2.5 (8.06)	NegBinom	[7, 24]
- Severe ($R_{0}^{s}$)	3.75 (11.22)	NegBinom	[28]
Diagnosed Individuals	69%	NA	[4]
- Severe cases	16%	NA	[38]
Population size	100	NA	-
Contact Rate (λ)	12/day	NA	[26]
Contact Rate quarantine (λ_*q*_)	3/day	NA	[26]
Contact Rate isolation (λ_*i*_)	0.6/day	NA	Assumed
Infectivity measure	5.76 (1.66)	Gamma	Assumed

The relative infectivity of a severe versus a mild case is set to *χ*_*s*_ = 1.5. Undiagnosed individuals are assumed to be less infectious than diagnosed and the relative infectivity is set to *χ*_*a*_ = 0.55 [4], while having the same infectious period length of mild cases [30, 34]. We assume the diagnosed individuals to be 69% of the total cases, in line with the reporting rate computed in [4, 35] when travel restriction were in place. The selected infectivity values lead to an overall basic reproduction number $R_{0}=2.3$ when control measures are not accounted for, in line with literature estimates for the COVID-19 pandemic [7, 21, 24]. This value varies in the considered scenarios according to the interventions considered. Moreover, the reproduction number is modeled according to a negative binomial distribution that accounts for multi-spreading events. The overdispersion parameter is set to be of value *k* = 0.16 in line with what in Lloyd et al. [36] for SARS. This value is in line with recent estimates of the overdispersion parameter for COVID-19 [37]. We assume the same overdispersion parameters for diagnosed and undiagnosed individuals. The list of the parameters and distribution used in the baseline scenario is summarized in Table 1, together with the references used to inform such values.

For all the simulated scenarios, we simulated outbreaks on a population of size *n* = 1000 and compute the mean and the variance of the serial and generation intervals realized in each single simulation. Simulations start with one index case and continue until no infectious individuals are present in the population. For all the generations that take place in a single simulation, we compute the serial interval for each infector-infectee pair in which both individuals show symptoms while the generation time is computed for all of the infector-infectee pairs. Afterwards, we keep track of the mean values and the standard deviation of such quantities realized in each simulation run. Last, we compute the average of the aforementioned quantities together with a simulation-based confidence interval to illustrate the simulation results. For each scenario, we simulate 100,000 outbreaks and we discard the ones in which less than 10% of the population is infected, to avoid that the estimates computed in a single simulation are affected by a limited number of generations.

### Simulation scenarios

We varied the time to isolation and the quarantine contact rate to investigate the efficacy of control measures. We first tested the effect of interventions that take place 1 day before symptom onset and after a gamma-distributed delay from symptom onset (average 3.8 days [39]). The first scenario is motivated by an epidemiological setting where a contact universal testing policy is implemented [40]. The second represents the early stage of the epidemic, where individuals were less aware of the disease. Furthermore, we look at the effect of the quarantine contact rate by assuming it is either λ_*q*_ = λ_*i*_ = 0.05λ or λ_*q*_ = 0.5λ.

Afterwards we investigate the impact of the undiagnosed population. We varied the prevalence of undiagnosed individuals accounting for a scenario in which all the infectives are diagnosed and another in which a big proportion of cases remains undetected (i.e. 86% of undiagnosed infectious individuals to represent the findings of Li et al. for the emerging phase of the pandemic [4]). Last, we looked at the impact of the infectivity of such undiagnosed infectious individual. We tested the setting in which asymptomatic cases are as infectious as individuals with a mild infection, and the setting in which their infectivity is a small fraction of the mild symptomatic one, i.e. 10%. The choices of these values are based, respectively, on the observation that mild symptomatic and asymptomatic individuals have a similar viral load [30, 34] and on the relative infectivity of asymptomatic individuals estimated in Ferretti et al. [41].

## Results

### Impact of control measures on the mean serial and generation intervals

**Fig 2:**
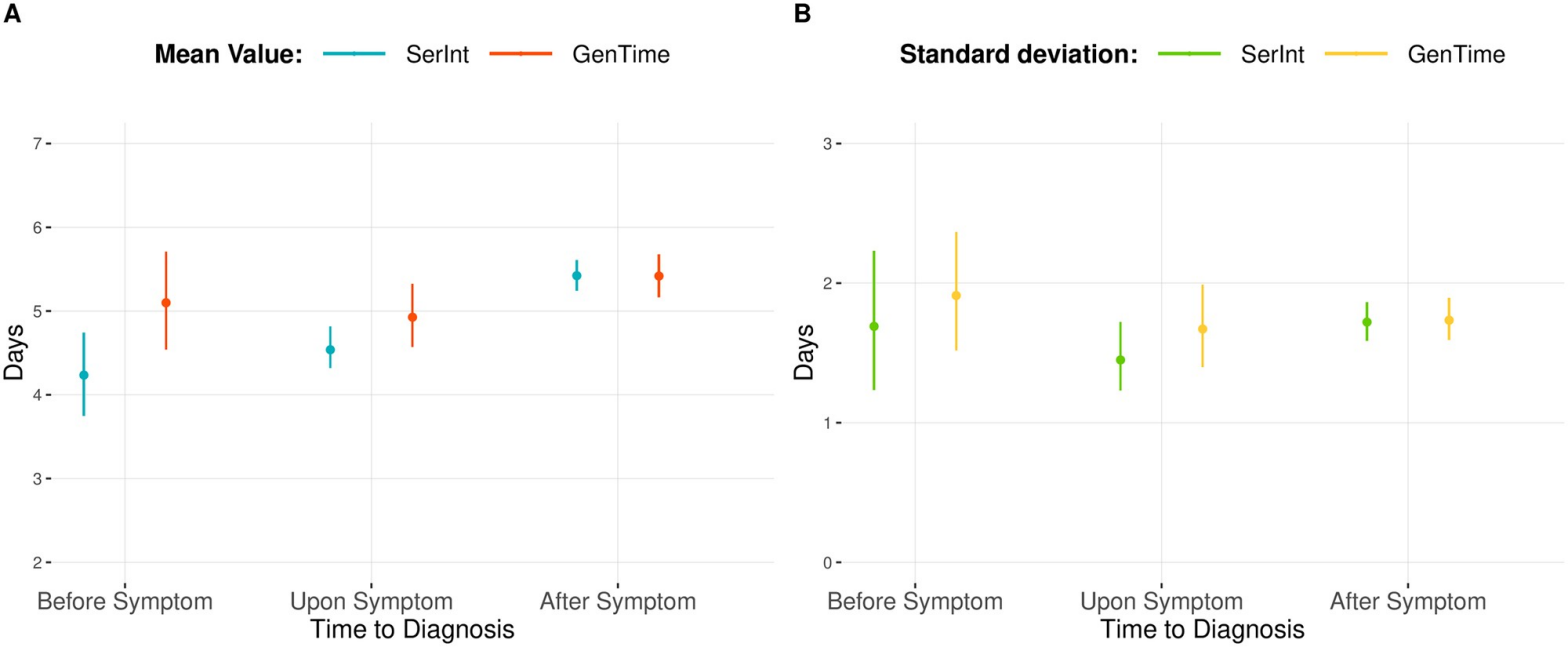
Control measure—Time to diagnosis. (A) Average and 95% confidence intervals for the mean realized serial and generation intervals and (B) associated realized standard deviations (B) for diagnosis before the onset of symptom (Before Symptom), coincident with symptom onset (Upon Symptoms) and gamma-distributed delays to diagnosis (Gamma distr.—Baseline).

The time to diagnosis affects both the serial and the generation intervals. The mean serial interval contracts accordingly to the start of interventions: the sooner quarantine or isolation starts, the shorter the mean serial interval (Fig 2). Similarly, the mean generation time contracts when interventions act upon or before the onset of symptoms. We note that the average generation time slightly increases when diagnosis takes place upon symptom respect to the Before Symptom scenario. This is due because of the proportion of generation times realized by diagnosed and undiagnosed individuals and by their mean generation time values. In fact, control measures decrease the contact rate, and consequently, the infectiousness of diagnosed individual. Therefore, the sooner intervention starts, the lower the proportion of cases generated by diagnosed individuals. Furthermore, the mean generation time for diagnosed cases, which is always shorter than the one of undiagnosed cases, increases when control measures start later on in the infectious period (Fig C in S1 Appendix). Therefore, the overall mean generation time results to be on average larger in the Upon Symptom scenario compare to the Before Symptom scenario. Moreover, we noticed that the mean serial interval is smaller than the mean generation interval when interventions start upon or before the onset of symptoms. In addition, this deviation depends on time to diagnosis. The standard deviation of both quantities also depends on control measures, with higher values when intervention act before the onset of symptoms. Moreover, when interventions take place before, or at the time of symptom onset, the generation time has on average a larger variation compare to the serial interval. When the quarantine contact rate varies, the mean and standard deviation of the serial interval decrease for a high contact reduction, while the mean and standard deviation of the generation time is approximately the same among the different scenarios (S1 Appendix).

### Proportion of undiagnosed infectives

**Fig 3:**
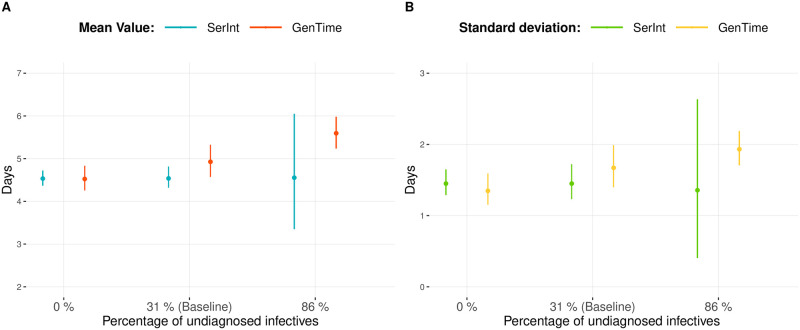
Undiagnosed population—Prevalence. (A) Average and 95% confidence intervals for the mean realized serial and generation intervals and (B) associated realized standard deviations when all the infectives are diagnosed (0%), 31% of infectives are not diagnosed (31%) and 86% of the infectives are not diagnosed.

The generation times realized by undiagnosed individuals increase the overall mean generation time when interventions are in place. In fact, such individuals, who are not affected by control measures, have a larger mean generation time compared to the generation time of diagnosed ones (Fig C in S1 Appendix). Undiagnosed individuals are more likely to make generations later on in their infectious period compared to the diagnosed ones, who are affected by interventions. This can be observed in Fig 3, where the prevalence of undiagnosed infectious individuals varies among: 0%, 31% and 86% reflecting limited test availability as observed early in the pandemic in several settings [4]. While the average serial interval is the same among the different simulated scenarios, the generation time increases according to the prevalence of undiagnosed infectives. The standard deviation of the serial interval is observed to be on average approximately the same. However, a high variation is observed when a high prevalence of asymptomatic individuals is considered, i.e. 86% undiagnosed. In the latter case, the high variation is caused by the limited number of generations registered between sypmtomatic individuals, which are far less likely to occur. Differently, the standard deviation of the generation time increases with the increasing prevalence of undiagnosed infectives. We noticed that the generation interval has a higher standard deviation compared to the serial interval except in the case in which all the individuals are diagnosed, i.e. scenario 0%.

Furthermore, we varied the reproduction number of the undiagnosed population comparing the cases: $R_{0}^{u}=0.1R_{0}^{d},$ $0.55R_{0}^{d},$ $R_{0}^{d}$ (S1 Appendix) when the 31% of infectives are undiagnosed. We noticed that the mean and standard deviation of the generation time for a lower relative infective of undiagnosed cases than mild symptomatic ones. Differently, mean and standard deviation of the serial interval are on average the same among the tested scenarios Undiagnosed individuals, who have a larger mean generation time respect to diagnosed case (Fig C in S1 Appendix), realize a higher proportion of generations for an increasing value of the reproduction number consequently increasing the generation interval. Furthermore, an increase of the undiagnosed reproduction number does not affect the realized mean serial interval, since these individuals do not contribute to the serial interval and the same time to diagnosis is here considered.

### Difference between mean serial and mean generation intervals

**Fig 4:**
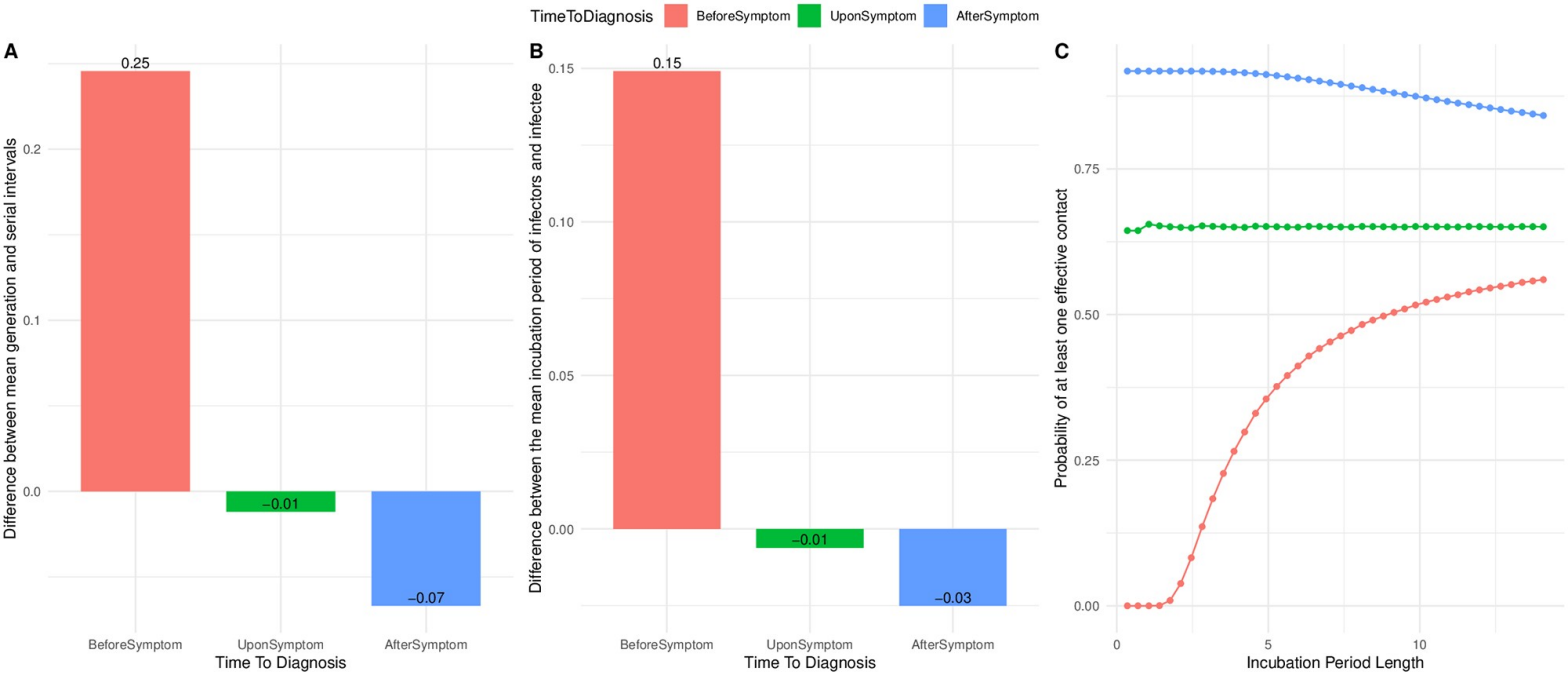
Difference between mean serial and generation intervals and realized incubation periods. (A) Difference between mean serial and generation interval and (B) between the realized incubation period of infectors and infectee when the time to diagnosis varies among: 1 day before symptom onset (BeforeSymptom), at symptom onset (UponSymptom), after a gamma-delay from symptom onset (AfterSymptom). (C) Probability that infectious individuals make at least one effective contact during their infectious period, for a varying incubation period length.

Results of the simulation study highlight that the mean serial and generation intervals do not coincide. While from one side this is caused by the presence of undiagnosed individuals, the time to intervention is also affecting the deviation between the two aforementioned values. To get more insight on this, we considered a scenario in which all the infectious individuals are diagnosed and we computed the difference between mean generation and serial intervals when the time of diagnosis varies (Fig 4A). Together with this, we reported the difference between the realized incubation period of infector, i.e. infectious individuals who make at least one generation during their infectious period, and infectee (Fig 4B). The two barplots show the same trend suggesting that the difference between serial and generation intervals is in line with the difference in the realized incubation periods of infectors and infectees.

Interestingly, the probability that infectious individuals with a specific incubation period length will make infections depends on the intervention measure in place, as shown in Fig 4C. In other words, control measures facilitate infectious individuals with a specific length of the incubation period in realizing generations, further leading to a deviation between the mean serial and generation intervals. We note that the result reported in Fig 4 reflects the theoretical description presented in section 2.1. In addition, we tested the effect of different parametrization of the infectivity measures, reported in S1 Appendix. The deviation between mean serial and generation intervals are qualitatively the same, but the magnitude seems to depend on the chosen implementation. This highlights the importance of the relationships between infectiousness, incubation period and time to interventions in the difference between mean realized serial and generation intervals.

## Discussion

With this investigation, we present an analysis of the effect of control measures on realized serial and generation intervals. In the following, we discuss the results obtained in this study in relation to the limitations and the assumptions made. Throughout the manuscript we consider aggregated values of generation and serial intervals. These values are affected by the depletion of susceptibles and the contraction can have a different magnitude on the generation and on the the serial intervals. However, we expect this population dynamic to possibly affect only the scenario “After Symptom” (Fig 2), since the effect of interventions reduces in the other scenarios the value of the individual reproduction number below 1.6, for which the effects of depletion of susceptibles are limited [22, 42]. The realized generation and serial intervals depend on the control measure in place. Their mean values decrease when interventions occur sooner in the infectious period and with a higher contact rate reduction during quarantine. In fact, because interventions limit social interactions, the probability of realizing a generation during quarantine or isolation decreases accordingly. This result is in line with Ali et. al and Sun et al. who described a shortening of the mean serial and generation intervals in relation to isolation [20, 21]. The standard deviations are also found to depend on the interventions in place, with a higher value for the generation time with respect to the serial interval when interventions act upon or before the onset of symptoms. To the best of our knowledge, there are not theoretical results on the effect of control measures on the variance of serial and generation intervals. The simulation study proposed in this manuscript is a first attempt to study such a relationship.

Furthermore, the presence of undiagnosed but infected persons is shown to affect the realized generation time. Its impact depends on the intrinsic generation time distributions of undiagnosed and diagnosed population, and on the effect of intervention measures on the latter (Fig C in S1 Appendix). In the presented investigation, we considered the same intrinsic generation time distribution for undiagnosed and mild cases, in line with viral shedding observation reported in Zhou et al. [30]. When this is the case, undiagnosed individuals increase the overall realized generation time. In the literature there are contrasting studies on the viral shedding of asymptomatic carriers compare to the one of symptomatic [31, 32, 35]. If this is longer in asymptomatic individuals, as argued in Long et al. [32], the impact on the realized mean generation time is expected to be larger, while if shorter, as reported in Kim et al. [31], the impact is expected to be smaller.

Interestingly, the observed mean serial and generation intervals are of different value, with the former shorter than the latter when control measures act upon, or before, the onset of symptom. This result depends on two factors. First, the presence of an undiagnosed population that has a larger mean generation time compared to the one of the diagnosed population. Therefore, undiagnosed individuals increase the realized generation time while not affecting the realized serial interval. Moreover, control measures favor the infectious individuals with a specific length of the incubation period to actually realize generations. As a consequence, we highlight the importance of the relationship between infectiousness, timing of intervention and incubation period in describing the generation and serial interval in a setting where interventions are in place. The serial interval has been argued to have higher variance compared to the generation time [17]. Here, we showed that the difference between the standard deviations of the aforementioned quantities depends on the control measure in place and on the prevalence of undiagnosed infectious individuals. In particular, in the tested settings the generation time has often larger standard deviation compare to the serial interval, specially when interventions take place before or upon the onset of symptoms and for a high prevalence of undiagnosed individuals. While this could be in part affected by pair selection, control measures are computed to contract differently serial and generation intervals. Consequently, their variances decrease with a different magnitude expressing that also the difference difference between these two values depend on the control measure in place. We notice that notwithstanding these issues, Ganyani et. al. found that estimated generation and serial intervals were approximately the same in (diagnosed) infection pair data from Tianjin and Singapore [3]. This is in line with the scenario presented in Fig 3A when considering only diagnosed individuals.

While the incubation period is on average of 5.2, in line with literature results [7, 24], its distribution is less skewed and concentrated around the mean value (Fig B in S1 Appendix). This directly depends on the model assumptions chosen to define the infectivity curve and how such curve is scaled among different infectious individuals. This bias possibly affects the magnitude of the reported difference between the mean serial and generation intervals. However, these two values are still expected to be different, since interventions possibly affect the incubation period of infectors and undiagnosed indivuals only contribute in increasing the mean generation time.

In our model, we parametrized the infectivity measure using a gamma density function (Fig 1) based on viral load observations. However, it is not clear yet whether virological data and infectiousness are related, and, if so, what is the underlying relationship among these quantities. Recently, Buonananno et al. presented an approach for estimating the viral load emitted by symptomatic and asymptomatic individuals. They applied such findings to infection risk models with the aim of explaining airborne transmission [43]. Even if further research is needed, this indicate that a relationship between infectiousness and viral load is plausible. Furthermore, the scaling procedure we used to assign the infectivity measure to the infectious individuals leads to a positive correlation between the length of the presymptomatic and symptomatic phases. In S1 Appendix we tested the impact of different parametrization of the infectivity measure for the same scaling procedure, but we did not notice particular differences with respect to the baseline case. To this regard, data in which is possible to estimate the correlation between the phases of infection, and the possible relationship between infectiousness and viral load would be of outmost importance for viral transmission model as the one presented in this work.

Information on the serial interval is often used as proxy of the generation time, e.g. in the estimation of the basic reproduction number, e.g. [10, 16]. This procedure has been argued to lead to underestimation bias, since the standard deviations are different [17]. In addition, we here argue that also the mean values are likely to be different, potentially increasing the estimation bias when using such an approximation. Recently, Park et al. developed a theoretical framework that links the serial interval to the reproduction number [19]. Their approach considers various sampling schemes, in particular forward and backward measurements and cohort based sampling, both for individual and pair-based durations, showing that the renewal process between symptomatic cases can be used to estimate *R*_0_. Their framework does not account for the contribution of asymptomatic individuals leading to possibly biased estimates when asymptomatic carriers and asymptomatic infections are plausible. Furthermore, their approach deals with the epidemic dynamics, not with the effects of interventions, as in this work. In conclusion, there is a need for a general theory of the relation between observation schemes, intrinsic dynamics and interventions, and estimates of fundamental epidemic parameters. An alternative approach to estimate the generation time and reproduction number could be based on the inference of the effective contact process. In fact, the rate of the effective contact process gives an approximation of the basic reproduction number and of the intrinsic generation interval distribution. To perform such inference, the underlying contact process needs to be known, the probability of infection given a contact, i.e. infectiousness, and the effect of the control measure. Several studies are present in literature describing social contact among individuals, e.g. [44], and infectiousness often described by viral load profile, e.g. [45]. If the relationships between viral load and infectiousness is established, an early estimate of the infectiousness profile could be inferred using social contact and viral load data.

In this work we focus on control measures that take place at an individual level. Furthermore, we assume that diagnosed individuals fully comply with quarantine measures until fully recovered. While this is in part captured by the prevalence of undiagnosed individuals and by the isolation and quarantine contact rate reductions tested, this could affect the overall generation and serial interval values. Other types of interventions which decrease infectiousness were in place at a population level, such as strict lockdown and promoting prevention measures as face-masks and social distancing. While these interventions were argued to decrease the reproduction number [13], their effect on the serial and generation times is still not certain. Ali et al. found a better fit to data when explaining the decrease of the mean serial interval if all these non-pharmaceutical interventions were jointly considered [20], suggesting a possible impact on realized serial and generation times. In addition, a strict lockdown was shown to increase the proportion of household infections [46], which can possibly be realized in shorter time [47] because of susceptible depletion. Therefore, the serial and generation time could be affected also by such population-level interventions.

## Supporting information

S1 Appendix Supplementary material. Additional information on model assumptions and description, and additional simulation scenarios.
(PDF)
