# Supplementary material for: On realized serial and generation intervals given control measures: The COVID-19 pandemic case
Source: PLoS Comput Biol. 2021 Mar 29;17(3):e1008892. doi: 10.1371/journal.pcbi.1008892 (PMC8031880; doi:10.1371/journal.pcbi.1008892)
Supplement: S1 Appendix — Additional information on model assumptions and description, and additional simulation scenarios. (PDF) [file pcbi.1008892.s001.pdf]

Supplementary Material

In this section we first explain in detail how infectivity is scaled among different infectious inidividuals, we report the incubation period distribution resulting from the model assumptions and we show the effect of isolation and quarantine on the intrinsic generation time distribution. Afterwards, we report the extended analysis for the contact rate during quarantine, the asymptomatic reproduction number and the extinction probability.

Infectivity measure, incubation period and generation time

In the simulation model we account for heterogeneity in the host viral progression. To do so, we first draw the length of the infectious period, μ_i, of an infectious individual, i. Then, we define the individual infectivity measure for individual i, ν_i, as:

$$\nu_i(t) = \frac{15}{\mu_i}\nu(\frac{15}{\mu_i}t)$$

where $\nu(t)$ is the infectivity curve defined in the method section (Table 1 and Fig 1). By means of such a scaling, the shape of the viral progression is the same among different infectious individuals. What varies is the length of the infectious period, μ_i that we model here using a gamma distribution with mean 15 days and a standard deviation of 2.7 days. We selected these shape and scale parameters so that the infectivity peaks on average after 5.2 days from infection and has positive value for 10 days on average. We did so, to represent literature findings on the COVID-19 pandemic [1–3]. Below, we report the progression of 40 infectivity measures set according to such a framework. The model assumptions lead to obtain an average incubation period of 5.2, in line with literature findigs. However, the incubation period distribution is less skewed and more concentrated around the mean value (Fig B), respect to what observed in research studies, e.g. Li et al. and Zhang et al. [1; 2]. This depends on the functional form selected for the infectivity measure, by the scaling of the infectivity measure and by the assumption that individuals show symptoms at the peak of such curve.
In our model, the generation time distribution depends on both the infectivity measure and the contact rate. Therefore, control measures, which reduce the contact rate, affect the generation time distribution. To show this effect, we report in Fig C the intrinsic generation time distribution considering isolation and quarantine for the times of intervention considered in this manuscript .

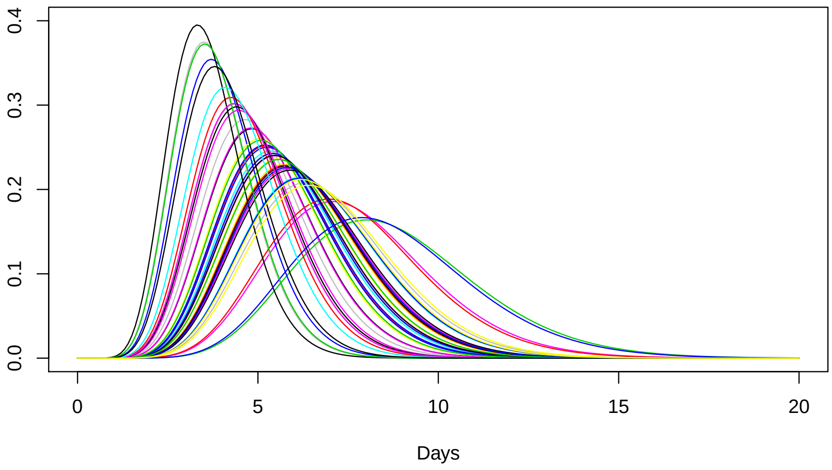

Fig A. Scaling the infectivity measure. Temporal progression of 40 infectivity measures randomly drawn.

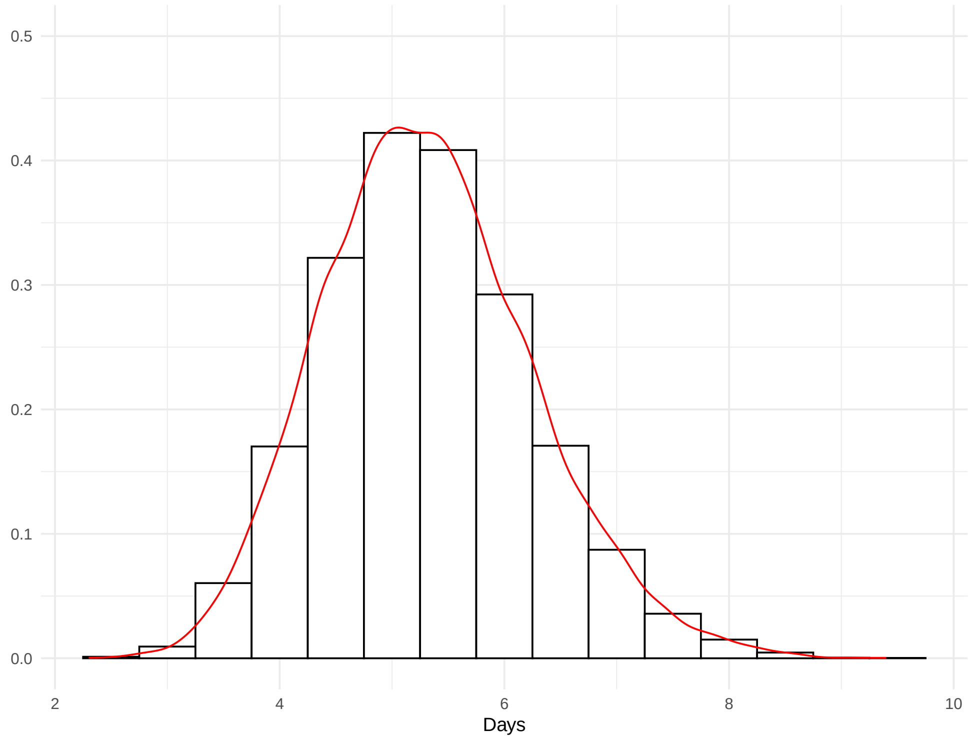

Fig B. Incubation period density. Smooth approximation of the incubation period density resulting from the model setting.

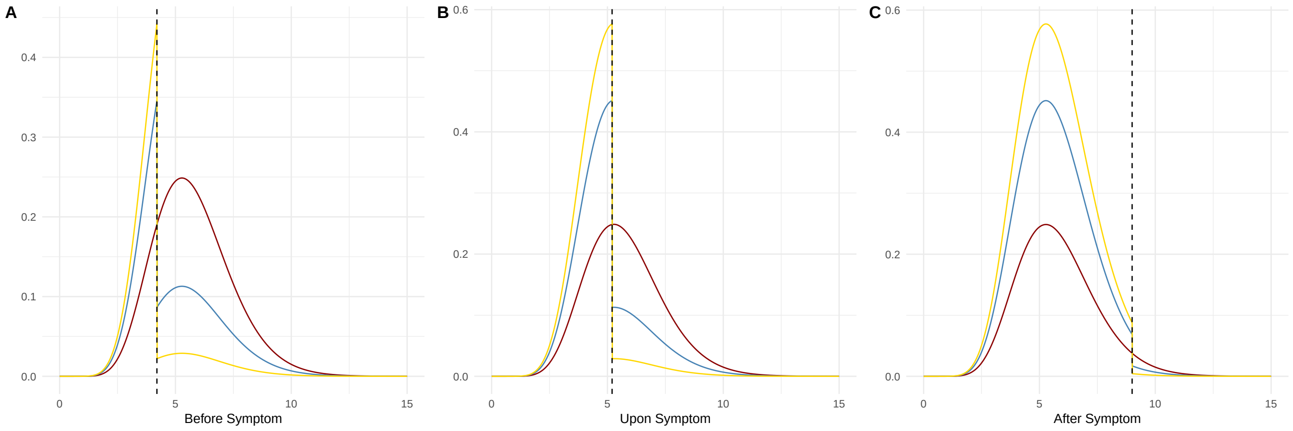

Fig C. Effect of control measures on the intrinsic generation time. Intrinsic generation time density when: no interventions are considered (dark red line), quarantine starts at the onset of symptom (blue line), isolation starts at the onset of symptom (yellow line) when interventions start before the onset of symptom (Before Symptom), upon symptom (Upon Symptom) and with a Gamma delay from thee onset of symptom (After Symptom). Vertical lines describe the start of the considered intervention

Dependence on the shape of the infectivity measure

We look at the effect of different viral progression shapes on the difference between mean serial and mean generation intervals. To do so, we select two other profiles described by a Weibull density function, in addition to the baseline scenario presented in Table 1. As shown in Fig D, we consider curves for which the onset of symptom coincide with the peak, to allow a comparison between them. In one case (Scen-A) the are below the curve is more concentrated before the onset of symptom, while in the other case (Scen-B) after. Similarly to analysis performed in the manuscript, we report the differences between

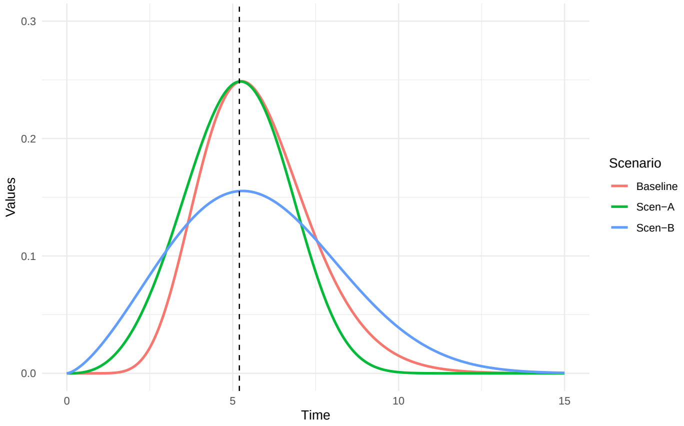

Fig D. Infectivity Measures. Temporal progression of the infectivity measures considered in this section.

mean serial and generation interval together with the difference between the realized

incubation periods of infectors and infectee. Results, for the "Scen-A" and "Scen-B" are reported, respectively in Figs E and F. We noticed similar results obtained for the

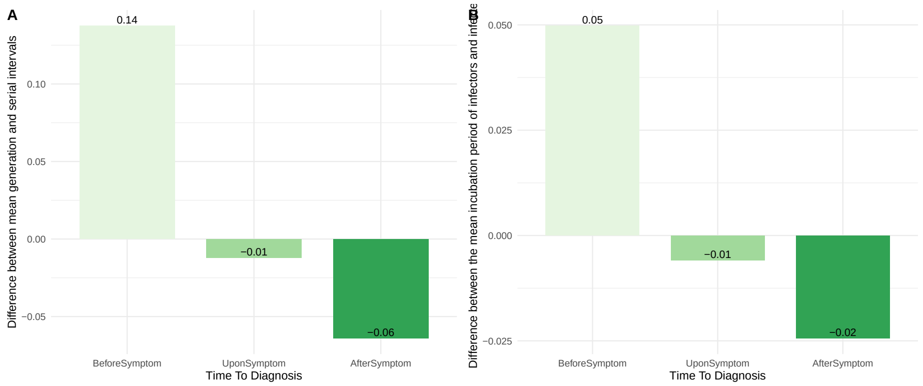

Fig E. Differences between serial and generation intervals and realized incubation periods.

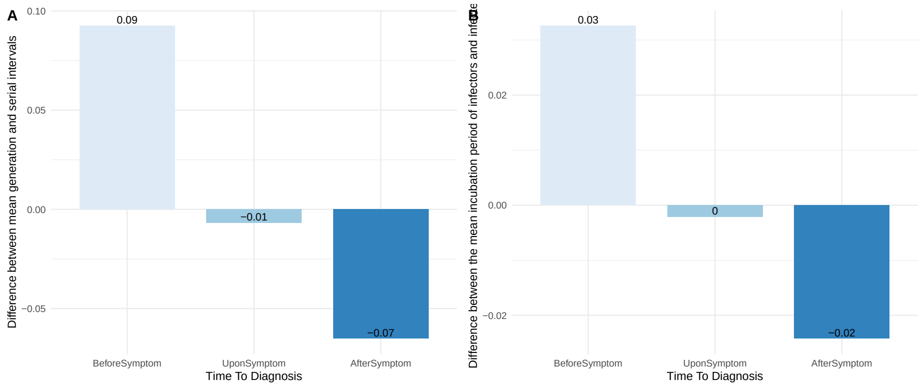

Fig F. Differences between serial and generation intervals and realized incubation periods.

baseline scenario (Fig 4). Though, the magnitude of the deviation differs between the different implementation.

Contact rate during quarantine

We vary the contact rate during quarantine, λ_q, to account for a different efficacy of the control measure. Next to baseline scenario, (Table 1) we considered $\lambda_q = \lambda_i = 0.6$ and $\lambda_q = 6$ contacts/day. As shown in Fig GA the decrease in the quarantine contact rate decreases the mean serial and generation intervals. Similarly, the standard deviations also increase according to the quarantine contact rate value (Fig GB). Both these summary measures increase because it is more likely to observe generation during quarantine when the quarantine contact rate is higher.

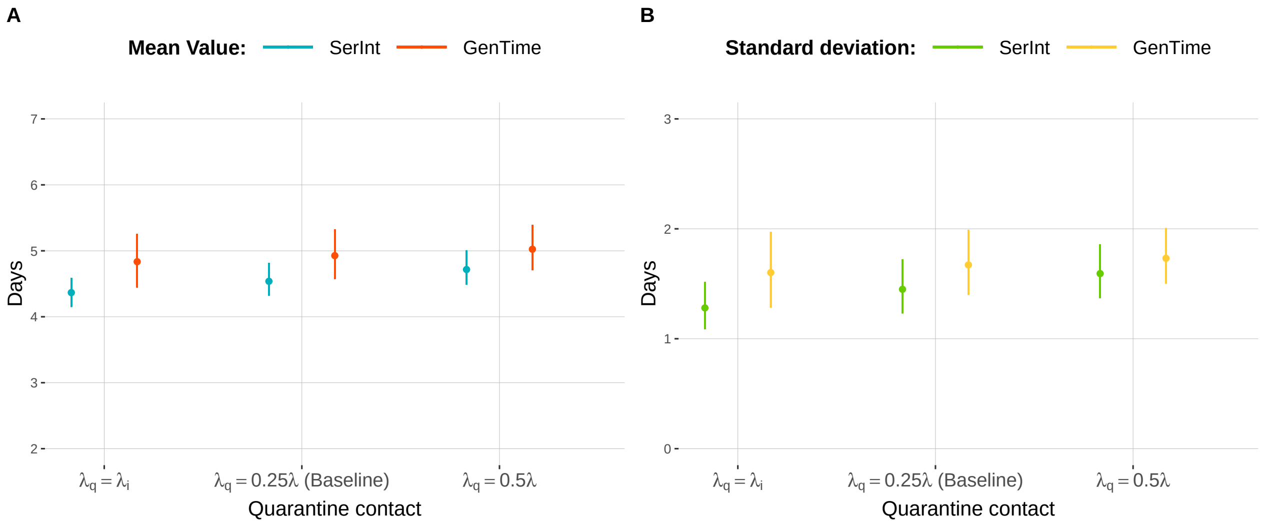

Figure 1: **Fig G. Quarantine contact rate.** (A) Average and 95% confidence intervals for the mean realized serial and generation intervals and (B) associated realized standard deviations when the quarantine contact rate is of value: $\lambda_q = \lambda_i, 0.25\lambda, 0.5\lambda$.

Undiagnosed Reproduction Number

In Fig H we reported the mean and the variance of realized serial and generation times when the reproduction number of the undiagnosed population varies. The tested values are $\mathcal{R}_0^u = 0.1\mathcal{R}_0^m, 0.55\mathcal{R}_0^m, \mathcal{R}_0^m$. We noticed that the mean generation time increases for a reproduction number bigger than one, since this guarantees, on average, that at least one generation is realized by undiagnosed individuals. Instead, we do not notice variation in the mean serial interval.

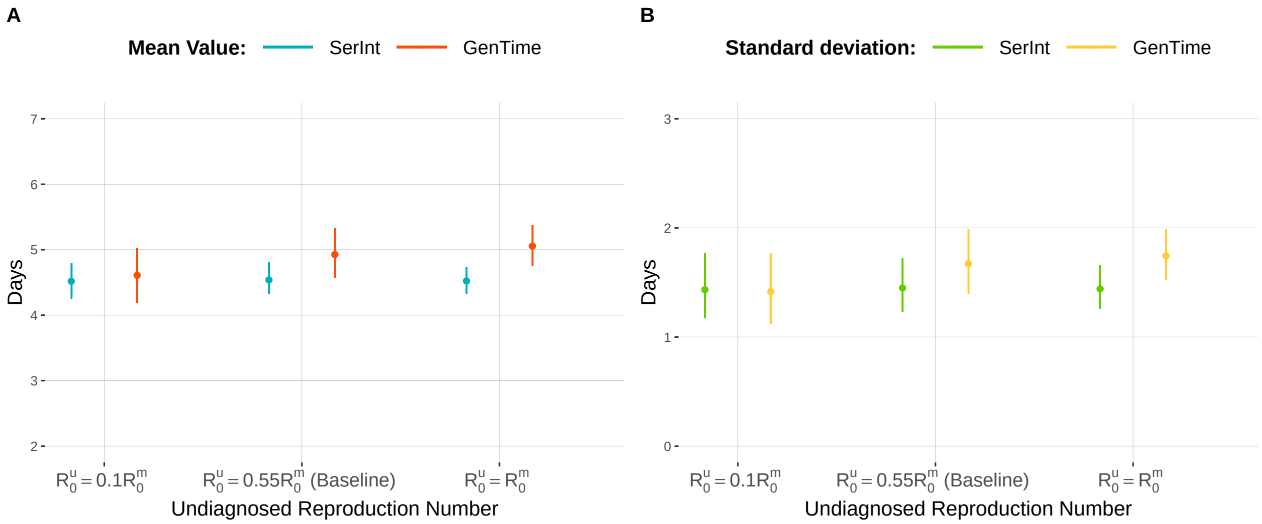

Fig H. Undiagnosed reproduction number. (A) Average and 95% confidence intervals for the mean realized serial and generation intervals and (B) associated realized standard deviations when the undiagnosed reproduction number is of value: $\mathcal{R}_0^u = 0.1\mathcal{R}_0^m, 0.55\mathcal{R}_0^m, \mathcal{R}_0^m$.

Extinction probability

Here, we consider the proportion of simulated outbreaks in which less than the 10% of individuals are ultimately infected. We assume this value to be an approximation of the extinction probability, i.e. the probability that an outbreak fades out without causing infections in a substantial fraction of the population. In Fig I we report the proportion of simulated outbreaks in which more than the 10% of individuals are ultimately infected, varying the time to diagnosis and the prevalence of undiagnosed infectives. The other parameters are set in line with the baseline scenario (Table 1).

The sooner interventions take place the lower the individual reproduction number results to be, consequently resulting in a higher extinction probability (Fig IA). Moreover, the extinction probability increases when the prevalence of undiagnosed infectives slightly increases (Fig IB). In general, this last result depends on the relative infectivity, i.e. $\frac{\mathcal{R}^u}{\mathcal{R}^u}$, while a similar impact of control measures is expected for all the interventions that reduce infectiousness.

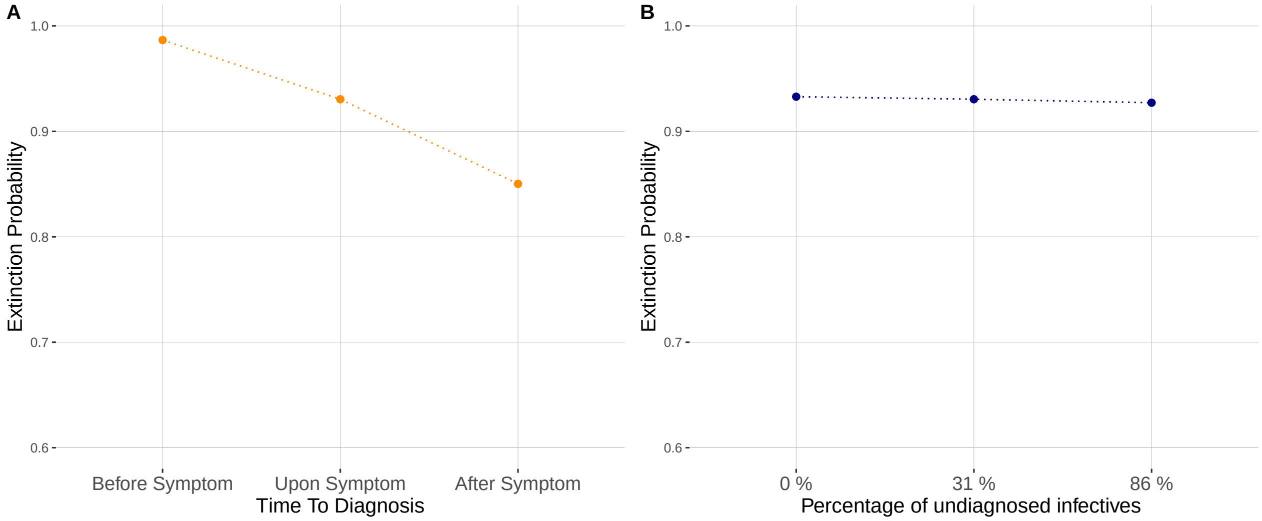

Fig I. Extinction Probability. Extinction probability value in scenarios for which the time to diagnosis (A) and the prevalence of undiagnosed infectives (B) vary.

Average mean and standard deviation values

In Table A we report the averages of the mean and standard deviation of realized serial and generation intervals among the simulated outbreaks for the different scenarios.

Table 1: **Table A. Averages of the mean and standard deviation values among the different scenarios.**

**Scenario**	**Mean (Sd) Serial Interval**	**Mean (Sd) Generation Interval**
Before Symptom	4.23 (1.69)	5.10 (1.91)
Upon Symptom	4.54 (1.45)	4.93 (1.67)
After Symptom	5.42 (1.72)	5.42 (1.73)
0 %	4.53 (1.45)	4.52 (1.38)
31 %	4.54 (1.45)	4.93 (1.67)
84 %	4.55 (1.36)	5.59 (1.93)
$\lambda_q = \lambda_i$	4.37 (1.28)	4.83 (1.60)
$\lambda_q = 0.25\lambda$	4.54 (1.40)	4.88 (1.54)
$\lambda_q = 0.5\lambda$	4.72 (1.59)	5.02 (1.73)
$R_0^u = 0.1R_0^m$	4.51 (1.40)	4.60 (1.32)
$R_0^u = 0.55R_0^m$	4.54 (1.40)	4.88 (1.54)
$R_0^u = R_0^m$	4.53 (1.40)	4.97 (1.61)

References

[1] Li Q, Guan X, Wu P, Wang X, Zhou L, Tong Y, et al. Early Transmission Dynamics in Wuhan, China, of Novel Coronavirus-Infected Pneumonia. New England Journal of Medicine. 2020;Available from: https://doi.org/10.1056/NEJMoa2001316.

[2] Zhang J, Litvinova M, Wang W, Wang Y, Deng X, Chen X, et al. Evolving epidemiology and transmission dynamics of coronavirus disease 2019 outside Hubei province, China: a descriptive and modelling study. The Lancet Infectious Diseases. 2020;Available from: https://doi.org/10.1016/S1473-3099(20)30230-9.

[3] Cevik M, Tate M, Lloyd O, Maraolo AE, Schafers J, Ho A. SARS-CoV-2, SARS-CoV, and MERS-CoV viral load dynamics, duration of viral shedding, and infectiousness: a systematic review and meta-analysis. The Lancet Microbe. 2020/12/22;Available from: https://doi.org/10.1016/S2666-5247(20)30172-5.
